# Supplementary material for: Common Complications of Sickle Cell Disease: A Simulation-Based Curriculum
Source: MedEdPORTAL. 2021 Apr 2;17:11139. doi: 10.15766/mep_2374-8265.11139 (PMC8034233; doi:10.15766/mep_2374-8265.11139)
Supplement: Supplementary file 1 — Case 1 - Acute Chest Syndrome.docxCase 2 - Stroke.docxCase 3 - Sepsis.docxSupplemental Images.docxCritical Action Checklists.docxDebrief Guide.docxPre- and Posttest.docx [file mep_2374-8265.11139-s001.zip › A. Case 1 - Acute Chest Syndrome.docx]

| **Appendix A: Simulation Case 1**  **SIMULATION CASE TITLE: “Acute Chest Syndrome in a Patient with Sickle Cell Disease”**  **AUTHORS: Cassondra Cramer-Bour MD, Justin Peterson MD, Barbara Walsh MD, Elizabeth S Klings MD**  **LEARNER AUDIENCE: post-graduate year 2 internal medicine residents** | |
| --- | --- |
| **PATIENT NAME: Ari Throcyte**  **PATIENT AGE: 24 years**  **CHIEF COMPLAINT: chest pain in the setting of vasoocclusive crisis**  **PHYSICAL SETTING: simulated hospital room** | |
|  | |
| **Brief narrative description of case** | *This case describes a 24-year-old man admitted to the general medical ward for vasoocclusive pain. He has worsening shortness of breath and chest pain reflective of developing acute chest syndrome. The learner goals are to recognize this complication of sickle cell disease and to manage it appropriately.* |
| **Primary Learning Objectives** | *• Recognize and appropriately manage acute chest syndrome leading to cardiopulmonary compromise*  *• Manage progressive hypoxemia with the available equipment*  *• Describe at least 1 etiologic factor of acute chest syndrome*  *• Execute appropriate management of acute chest syndrome including simple or exchange transfusion*  *•Obtain key diagnostics (ABG, CBC, LFTs, glucose, type & screen, reticulocyte percentage, cultures, chest radiograph (CXR))*   - *Demonstrate team work and communication skills by:* - *Appointing a team leader* - *Team leader assigns clear roles and tasks to available team members* - *Team utilizes closed-loop communication as appropriate* - *Team leader articulates a clear differential diagnosis* - *Team communicates in an open and respectful manner* |
| **Critical Actions** | 1. Describe an appropriate differential diagnosis for respiratory compromise in a patient with sickle cell disease 2. Appropriately treat the patient with escalating amounts of oxygen 3. Obtain key labs: arterial blood gas, lactate, CBC, type and screen 4. Obtain a portable chest radiograph and EKG 5. Diagnose acute chest syndrome 6. Reassess patient, recognize hypoxia is worsening and consider non-invasive ventilation vs mechanical ventilation 7. Order antibiotics, describe need for atypical coverage 8. Recognize need for blood transfusion (simple vs exchange transfusion – exchange will require hemodialysis catheter) 9. Consider fluid balance, consider diuretics with transfusion 10. Describe need for ICU and hematology consults |
| **Learner Preparation or Prebrief** | Patient is a 24-year-old man admitted to the adult floor for a vasoocclusive crisis (VOC). He is receiving IV D5 ½ NS at 200 ml/hour, intravenous morphine sulfate for pain control, and subcutaneous heparin for DVT prophylaxis. He has significant chest pain and even though incentive spirometry is ordered, he is not using it. The floor nurse pages the resident to tell them that the patient has a fever of 101.4F with an oxygen saturation of 84% on room air. The nurses have placed the patient on 3 liters via nasal cannula with improvement. |

| Initial Presentation | | | |
| --- | --- | --- | --- |
| **Initial vital signs** | T 101.4F; HR 138; BP 150/90; RR 24; O_2_ sat 93% on 3 liters NC | | |
| **Overall Setting and Appearance** | *Adult patient who appears uncomfortable but is alert and oriented. Exam is tachycardic, with a grade II systolic ejection murmur at the left upper sternal border, lung exam notable for crackles at bases bilaterally and occasional rhonchi.* | | |
| **Confederates (e.g., standardized participants) and their roles in the room at case start** | *The patient is a simulation mannequin* | | |
| **HPI** | The floor nurse pages the resident to tell them that the patient has a fever of 101.4F and that the O_2_ sat is 84% on room air. The nurse has placed the patient on 3 liters NC with improvement. The patient may respond to questions appropriately, stating she feels short of breath, fatigued and has substernal chest pain which is worsening over the last couple of hours. | | |
| **Past Medical/Surgical History** | **Medications** | **Allergies** | **Family History** |
| HbSS disease:   1. Hospitalized 2-3 times per year for VOC 2. History of ACS x 1-2 3. No prior history of stroke, AVN, DVT/PE | Hydroxyurea, folic acid, IV morphine sulfate, ibuprofen, acetaminophen | No known drug allergies | Mother has sickle cell disease |
| **Physical Examination** | | | |
| **General** | Appears uncomfortable and alert, coughing | | |
| **HEENT** | No pharyngeal exudate, mucous membranes moist | | |
| **Neck** | No elevation in jugular venous pulse | | |
| **Lungs** | Bibasilar crackles which do not clear with cough, occasional rhonchi scattered throughout, mild respiratory distress | | |
| **Cardiovascular** | Tachycardic with II/VI systolic ejection murmur at LUSB, Normal S1 and S2, no edema | | |
| **Abdomen** | Soft, non-tender, non-distended | | |
| **Neurological** | No focal neuro deficit, CN 2-12 intact | | |
| **Skin** | Skin is flushed and warm, mild diaphoresis, no obvious skin breakdown | | |
| **GU** | Not assessed | | |
| **Psychiatric** | Appears anxious | | |

| Instructor Notes - Changes and CASE Branch Points  *This section should be a list with detailed description of each step than may happen during the case. If medications are given, what is the response? Do changes occur at certain time points? Should the nurse or other participant prompt the learners at given points? Should new actors or participants enter, and when? Are there specific things the patient will say or do at given times? There are a few examples given, but it is expected that most cases will have many more changes and potential branch points.*  *If you have a more complex branching algorithm than can be accommodated by the structure below, feel free to replace this section with your own. Look at some recent simulation publications on MedEdPORTAL for examples.* | | |
| --- | --- | --- |
| **Intervention / Time point** | **Change in Case** | **Additional Information** |
| *Patient is placed on monitor*  *Patient is examined* | *Patient responds to learner questions and provides history described above* | *Labs, cultures & imaging may be ordered but results not yet available*  *Antibiotics may be ordered* |
|  | *SpO2 will improve to 95% if placed on 100% non-breather, HR will improve to 115 if placed on 100% non-rebreather* | *Patient may state, “My breathing feels better,” if placed on 100% non-rebreather*  *EKG (if requested) will show sinus tachycardia rate 120s, no ST changes* |
| *Five minutes after start of case (or sooner if 100% non-rebreather is not utilized)* | *Patient becomes progressively dyspneic, respiratory rate increases, oxygen saturation drops to 70% on room air (83% on 100% non-rebreather), HR climbs to 152, repeat Blood pressure 158/98* | *Patient may state, “Doc it’s getting really hard to breathe”* |
| *Learners reassess patient* | *Rales bilaterally over lower half of lung fields, moderately severe respiratory distress* | *If a CXR was requested, it demonstrates a multifocal pneumonia with pulmonary vascular congestion*  *Labs return: ABG: pH 7.37 CO2 37 O2 60 on 100% non-rebreather, CBC - WBC 14.6 no./mm3, Hb 7.2 g/dL (baseline 8-9), Platelets 475,000 no./mm3 reticulocyte count 4%, bilirubin 3.2 mg/dL, LDH 445 U/L. Lactic Acid 3.6 mg/dL* |
| *Learners may order IV furosemide* | *Continued respiratory distress but with improved O_2_ sat to 88% on 100% non-rebreather (75% RA)* | *Patient may state, “I have to go to the bathroom”* |
| *Learner may attempt non-invasive ventilation (NIV)* | *No change* | *Patient will continue to be restless, will appear cyanotic* |
| *Learners should state diagnosis* |  | *Facilitator may prompt code leader to make diagnosis if not already stated* |
| *Either after NIV has been attempted or 8 minutes after start of case* | *Patient continues to deteriorate (RR increases to 32, O_2_ Sat 60%, HR 158, patient minimally responsive)* |  |
| *Learners may call for intubation* | *Patient improves, RR decreases to 22, O2 sat 99%, HR decreases to 115, BP 121/85* | *Intubation performed per hospital protocol* |
| *Learners should recognize need for exchange vs simple transfusion* |  | *Learner may call for Hematology consult* |
| *Sign out given to ICU accepting physician* |  | *Code Leader should give a short sign out to ICU physician* |
|  |  |  |

Ideal Scenario Flow:

Learners are prompted by the floor nurse that this patient is newly febrile, hypoxic and tachycardic. They should urgently respond and begin with taking a history, examining the patient and forming a differential diagnosis. The hypoxia should prompt rapid treatment, which will initially improve on 100% non-rebreather but will ultimately slowly decline throughout the case. On exam, the learner should recognize respiratory distress with some signs of mild volume overload. A chest x-ray should be performed, which will reveal the diagnosis of a multifocal pneumonia and mild pulmonary vascular congestion. Diuretics can be administered with mild relief. Antibiotics should be ordered (after blood cultures collected) with coverage for atypical organisms and encapsulated bacteria. The team leader should make a diagnosis of acute chest syndrome and call for assistance of Hematology. There should be a plan for simple and/or exchange transfusion. A simple transfusion is appropriate if the hemoglobin is significantly below baseline or if there is delay in arranging an exchange transfusion. If an exchange transfusion is recommended, the learner should discuss the need for hemodialysis catheter placement. Finally, the patient’s respiratory distress will prove refractory to non-invasive ventilation and will require intubation, which should be performed within the guidelines of the hospital. Following intubation, the patient should be transferred to the ICU and a brief practice sign out should be given to the accepting physician.

Anticipated Management Mistakes:

1. The learners may not recognize the need to place the patient on a 100% non-rebreather right away. Should there be a delay, the patient will more rapidly decompensate, the technical facilitator may voice (as the patient) “Doc, it’s getting really hard to breathe”, which should prompt more urgent management.
2. The learners may not correctly identify the case as “acute chest syndrome”, if this should happen, the diagnostic criteria can be reviewed in the debrief session.
3. Learners may not identify the need for simple vs exchange transfusion in the treatment of acute chest syndrome, this can also be discussed further in the debrief session
