# Supplementary material for: Common Complications of Sickle Cell Disease: A Simulation-Based Curriculum
Source: MedEdPORTAL. 2021 Apr 2;17:11139. doi: 10.15766/mep_2374-8265.11139 (PMC8034233; doi:10.15766/mep_2374-8265.11139)
Supplement: Supplementary file 1 — Case 1 - Acute Chest Syndrome.docxCase 2 - Stroke.docxCase 3 - Sepsis.docxSupplemental Images.docxCritical Action Checklists.docxDebrief Guide.docxPre- and Posttest.docx [file mep_2374-8265.11139-s001.zip › B. Case 2 - Stroke.docx]

| **Appendix B: Simulation Case 2**  **SIMULATION CASE TITLE: “Stroke in a Patient with Sickle Cell Disease”**  **AUTHORS: Cassondra Cramer-Bour MD, Justin Peterson MD, Barbara Walsh MD, Elizabeth S Klings MD**  **LEARNER AUDIENCE: Post-graduate year 2 internal medicine residents** | |
| --- | --- |
| **PATIENT NAME: Sid Culled**  **PATIENT AGE: 23 years**  **CHIEF COMPLAINT: headache in the setting of vasoocclusive crisis**  **PHYSICAL SETTING: simulated hospital room** | |
|  | |
| **Brief narrative description of case** | *This case describes a 23-year-old man admitted to the general medical ward for vasoocclusive pain. He complains of a headache and quickly decompensates with a hemorrhagic stroke. The learner is tasked with making this diagnosis and anticipating next steps with management of this condition.* |
| **Primary Learning Objectives** | *• Develop an appropriate differential diagnosis for an acute alteration in mental status in a patient with sickle cell disease*  *• Recognize and appropriately manage hemorrhagic stroke leading to neurologic compromise*  *• Obtain key diagnostics (ABG, CBC, LFTs, glucose, type & screen, reticulocyte count, cultures, CT head without contrast)*  *• Discuss role for anti-coagulation reversal agents*  *• Execute appropriate management of stroke with simple or exchange transfusion*   - *Demonstrate team work and communication skills by:* - *Appointing a team leader* - *Team leader assigns clear roles and tasks to available team members* - *Team utilizes closed-loop communication as appropriate* - *Team leader articulates a clear differential diagnosis* - *Team communicates in an open and respectful manner* |
| **Critical Actions** | 1. Describe an appropriate differential diagnosis for altered mental status 2. Perform a full neurologic exam 3. Obtain key labs: arterial blood gas, lactate, CBC, type and screen and coagulation studies 4. Recognize need for transfusion, simple vs exchange 5. Obtain a CT head without contrast 6. Diagnose hemorrhagic stroke 7. Reassess patient, recognize obtundation and need for intubation for airway control 8. Discuss role of reversal agents for anti-coagulation 9. Describe need for an ICU, hematology, neurology and neurosurgery consult |
| **Learner Preparation or Prebrief information** | Patient is a 23-year-old man who was admitted to the adult floor for a vasoocclusive crisis (VOC). He is receiving IV D5 ½ NS at 125 ml/hr, IV morphine sulfate and subcutaneous unfractionated heparin for DVT prophylaxis. |

| Initial Presentation | | | |
| --- | --- | --- | --- |
| **Initial vital signs** | T 98.8 F, HR 135, BP 140/70, RR 12, Oxygen saturation 95% on RA | | |
| **Overall Setting and Appearance** | *Adult patient who appears altered, lying in bed, requires frequent verbal cues to respond to questioning.* | | |
| **Confederates (e.g., standardized participants) and their roles in the room at case start** | *The patient is a simulation mannequin* | | |
| **HPI** | The floor nurse pages the resident and states that the patient was complaining of a headache earlier and now appears less alert. The patient reports a headache that started earlier in the day but has gotten worse, requires frequent prompting to answer questions, not able to describe headache, non-cooperative with exam. | | |
| **Past Medical/Surgical History** | **Medications** | **Allergies** | **Family History** |
| HbSS disease:   1. Hospitalized 2-3 times per year for VOC 2. History of ACS x 1-2 3. Childhood history of stroke (no residual deficit) | Hydroxyurea, folic acid, IV morphine sulfate, ibuprofen, acetaminophen, unfractionated heparin subcutaneous | No known drug allergies | Mother with sickle cell disease |
| **Physical Examination** | | | |
| **General** | Lying in bed, sleepy and drifts off during questioning, complains of a headache | | |
| **HEENT** | No pharyngeal exudate, moist mucous membranes | | |
| **Neck** | No jugular venous distension | | |
| **Lungs** | Clear, shallow even respirations at a rate of 10 | | |
| **Cardiovascular** | RRR, III/IV systolic ejection murmur at LUSB, Normal S1, S2, no edema | | |
| **Abdomen** | Soft, non-tender, non-distended | | |
| **Neurological** | Pupils are pinpoint, symmetric and sluggishly reactive. Unclear if any focal weakness or sensory deficit but LUE and LLE seem subtlety weaker than right side, effort on exam is poor. | | |
| **Skin** | Unremarkable | | |
| **GU** | Not assessed | | |
| **Psychiatric** | Sleepy, oriented to person, place and time but slow to answer | | |

| Instructor Notes - Changes and CASE Branch Points  *This section should be a list with detailed description of each step than may happen during the case. If medications are given, what is the response? Do changes occur at certain time points? Should the nurse or other participant prompt the learners at given points? Should new actors or participants enter, and when? Are there specific things the patient will say or do at given times? There are a few examples given, but it is expected that most cases will have many more changes and potential branch points.*  *If you have a more complex branching algorithm than can be accommodated by the structure below, feel free to replace this section with your own. Look at some recent simulation publications on MedEdPORTAL for examples.* | | |
| --- | --- | --- |
| **Intervention / Time point** | **Change in Case** | **Additional Information** |
| *Patient is placed on monitor* | *Patient responds to learner questions and provides history described above* | *Labs, cultures & imaging may be ordered but results not yet available*  *Antibiotics may be ordered* |
| *Patient is examined* |  | *Patient may state, “My head hurts”, or “I don’t feel so good”* |
| *Learner performs a full neurologic exam* | *Pupils are pinpoint with diminished respiratory effort, LUE and LLE seem weaker compared to right side but effort limited* | *Facilitator should prompt team leader for differential diagnosis if not already provided. A CT head may be performed but result not immediately available.* |
| *Learners may request naloxone* | *Respiratory effort improves, pupils are now dilated 4mm and symmetric, but the patient doesn’t become more alert* |  |
| *Within 5 minutes of the start of simulation* | *HR 110, BP 150/98, RR 12, O_2_ sat 88% on RA*  *Patient vomits and becomes obtunded, now with prominent LUE and LLE weakness* | *Labs return (if requested) : ABG: pH 7.37 CO2 37 O2 85 Bicarb 22, WBC 12 no./mm3, Hb 6.5 g/dL (baseline 7), platelets 535K no./mm3, blood glucose 92 mg/dL, INR 1.3, PTT 24 s, Lactate 2.0 mg/dL, AST 32 U/L, ALT 45 U/L, T bili 0.6 mg/dL, Alkaline Phosphatase 154 mg/dL* |
| *Learner should call for intubation* | *No change* | *Patient intubated per hospital policy*  *Of note, at this point a CT should not be obtained until after patient is intubated and stabilized* |
| *Learner may consider transfusion, either simple vs exchange (if exchange patient will need a hemodialysis catheter)* |  | *CT Head (if already requested) result: intracerebral hemorrhage emanating from the right MCA with extension into the lateral ventricle.* |
| *Learner should state diagnosis* |  | *Facilitator may prompt code leader to make diagnosis if not already stated* |
| *Learner may call for consultation of ICU, hematology, neurology and/or neurosurgery* | *Patient develops a left sided facial droop and unreactive left pupil* |  |
| *Learner should discuss holding subcutaneous heparin and consider reversal agents* |  |  |
| *Sign out given to ICU accepting physician* |  | *Code Leader should give a 1 liner sign out to ICU physician* |
|  |  |  |

Ideal Scenario Flow:

The learners enter the room and find the patient with severely altered mental status. They perform a full neurologic exam and consider a wide differential diagnosis including opioid overdose, meningitis, complex migraine headache and stroke. They should understand that patients with sickle cell disease are at risk for ischemic and hemorrhagic strokes. They can consider naloxone administration to counteract the opioids the patient is receiving for pain management but, ultimately, the patient decompensates further and is diagnosed with a large hemorrhagic stroke with extension of blood into the ventricles. A head CT without contrast is necessary to make the diagnosis. The patient should be intubated for airway control. The patient should receive a simple transfusion acutely to lower the hemoglobin S percentage, ideally as soon as stroke becomes the most likely diagnosis. Once the diagnosis of hemorrhagic stroke is made, all anticoagulants should be held and a discussion ensue regarding reversal agents if necessary. Consultation will be required of the ICU team, hematology, neurology as well as neurosurgery to consider ventricular drainage. The case ends with the team leader giving a practice “short sign out” to the accepting ICU physician.

Anticipated Management Mistakes:

1. The learners may overlook performing a full neurologic examination and make a delayed observation of subtle focal neurologic deficits. Should this happen the facilitator may suggest the team re-examine the patient and perform a full neurologic examination.
2. From our experience, many learners are not familiar with the indications for simple vs exchange transfusions in the management of stroke in patients with sickle cell disease. It is recommended to highlight this discussion in the debrief session.
3. The learners may overlook the detail that this patient was receiving prophylaxis with unfractionated heparin. Further, they may not recognize the need to order a type and screen with coagulation studies (INR/PT and PTT). If, during the simulation, there is no mention of anti-coagulation or the need for reversal agents, this will need to be discussed in the de-briefing session.
