# Supplementary material for: Common Complications of Sickle Cell Disease: A Simulation-Based Curriculum
Source: MedEdPORTAL. 2021 Apr 2;17:11139. doi: 10.15766/mep_2374-8265.11139 (PMC8034233; doi:10.15766/mep_2374-8265.11139)
Supplement: Supplementary file 1 — Case 1 - Acute Chest Syndrome.docxCase 2 - Stroke.docxCase 3 - Sepsis.docxSupplemental Images.docxCritical Action Checklists.docxDebrief Guide.docxPre- and Posttest.docx [file mep_2374-8265.11139-s001.zip › C. Case 3 - Sepsis.docx]

| **Appendix C: Simulation Case 3**  **SIMULATION CASE TITLE: “Sepsis in a Patient with Sickle Cell Disease”**  **AUTHORS: Cassondra Cramer-Bour MD, Justin Peterson MD, Barbara Walsh MD, Elizabeth S Klings MD**  **LEARNER AUDIENCE: Post-graduate year 2 internal medicine residents** | |
| --- | --- |
| **PATIENT NAME: Annie Mia**  **PATIENT AGE: 48 years**  **CHIEF COMPLAINT: fever in the setting of vasoocclusive crisis**  **PHYSICAL SETTING: simulated hospital room** | |
|  | |
| **Brief narrative description of case** | *This case describes a 48-year-old woman admitted to the general medical ward for vasoocclusive pain and fevers. She quickly decompensates with septic shock from vertebral osteomyelitis. The learners are tasked with recognizing sepsis and managing it in an immunocompromised host with appropriate antibiotic choices and judicious fluid resuscitation.* |
| **Primary Learning Objectives** | *• Develop an appropriate differential diagnosis for back pain and fever*  *• Recognize and appropriately manage sepsis in an asplenic patient*  *• Obtain key diagnostics (ABG, CBC, LFTs, glucose, T&S, reticulocyte count, cultures, UA, CXR and lumbar spine radiograph)*  *• Order broad spectrum antibiotics with coverage for encapsulated organisms*  *• Begin vasopressors after judicious fluid resuscitation, recognize these patients may have some degree of diastolic dysfunction and right ventricular compromise*   - *Demonstrate team work and communication skills by:* - *Appointing a team leader* - *Team leader assigns clear roles and tasks to available team members* - *Team utilizes closed-loop communication as appropriate* - *Team leader articulates a clear differential diagnosis* - *Team communicates in an open and respectful manner* |
| **Critical Actions** | 1. Describe an appropriate differential diagnosis for back pain and fever 2. Perform a head to toe exam looking for evidence of infection 3. Identify bony tenderness over lumbar spine using palpation 4. Obtain key labs: arterial blood gas, lactate, CBC with differential, BMP, LFTs blood cultures, UA and urine cultures. 5. Recognize skin breakdown associated with previous port-a-cath placement as a potential source of infection 6. Choose broad spectrum antibiotics with coverage for encapsulated organisms 7. Ensure patient has adequate access for fluid resuscitation, consider central line placement for vasopressors 8. Discuss an appropriate fluid resuscitation plan recognizing possible diastolic dysfunction and right ventricular compromise given longstanding sickle cell disease 9. Consider additional imaging to make diagnosis (ie CT or MRI of lumbar spine) 10. Call for ICU consult given need for vasopressors |
| **Learner Preparation or Prebrief** | 48 y.o. woman with history of HbSS disease admitted with fever, backache, lower extremity pain /low back pain typical of a vasoocclusive crisis. Fever to 103F noted 2 days prior to admission, resolved temporarily with ibuprofen, her back pain started at the same time and is consistent with past VOCs. She denies cough, dyspnea, chest pain, and dysuria. She did note some swelling associated with her port-a-cath placement which started around the same time. |

| Initial Presentation | | | |
| --- | --- | --- | --- |
| **Initial vital signs** | T 102.8 F, HR 155, BP 103/62, RR 25, SpO2 92% on RA | | |
| **Overall Setting and Appearance** | *Adult patient who appears very uncomfortable, writhing around in bed in pain. She is diaphoretic and anxious, keenly alert lying in her hospital bed.* | | |
| **Confederates (e.g., standardized participants) and their roles in the room at case start** | *The patient is a simulation mannequin* | | |
| **HPI** | *The floor nurse pages the resident to tell them the patient is febrile, tachycardic, borderline hypotensive and writhing around the bed in pain. The patient reports worsening back pain. She denies any worsening cough, dysuria or N/V/D/C.* | | |
| **Past Medical/Surgical History** | **Medications** | **Allergies** | **Family History** |
| HbSS disease, multiple admissions for VOC in past, asthma since childhood. Prior DVT/ PE. Long standing port-a-cath—removed and changed last admission 3 months prior for Staph epi bacteremia. | Oxycontin 10mg BID, oxycodone 60-90mg po q4 prn pain, Hydroxyurea | No known drug allergies | Sickle cell disease in mother |
| **Physical Examination** | | | |
| **General** | Ill appearing woman, diaphoretic and anxious | | |
| **HEENT** | No pharyngeal exudate, mucus membranes moist | | |
| **Neck** | No elevated JVD, neck is supple without rigidity | | |
| **Lungs** | Chest clear with shallow breaths due to back pain and splinting, No rales | | |
| **Cardiovascular** | Tachycardic, systolic murmur at LSB (old), Normal S1, S2, no edema | | |
| **Abdomen** | Soft, non-tender, non-distended | | |
| **Neurological** | No focal neuro deficit | | |
| **Skin** | R sided chest wall with port-a-cath in situ, slight tenderness to palpation and warmth, skin is flushed and diaphoretic | | |
| **MSK** | Good range of motion of bilateral hips, lumbar spine tender over L2-L4 with palpation. No CVA tenderness. | | |
| **Psychiatric** | Anxious appearing | | |

| Instructor Notes - Changes and CASE Branch Points  *This section should be a list with detailed description of each step than may happen during the case. If medications are given, what is the response? Do changes occur at certain time points? Should the nurse or other participant prompt the learners at given points? Should new actors or participants enter, and when? Are there specific things the patient will say or do at given times? There are a few examples given, but it is expected that most cases will have many more changes and potential branch points.*  *If you have a more complex branching algorithm than can be accommodated by the structure below, feel free to replace this section with your own. Look at some recent simulation publications on MedEdPORTAL for examples.* | | |
| --- | --- | --- |
| **Intervention / Time point** | **Change in Case** | **Additional Information** |
| *Patient is placed on monitor* | *Patient responds to learner questions and provides history described above* | *Labs, cultures & imaging may be ordered but results not yet available*  *Antibiotics may be ordered* |
| Patient is examined |  | *Patient may state, “Ouch” if R chest is palpated.* |
| *Learner may log roll patient to fully examine back* | *Patient will cry out in pain when log-rolled and back palpated.* | *Patient reports difficulty in urinating if asked, no bowel dysfunction* |
| *Learner may place patient on nasal cannula* | *SpO2 increases to 95% on 2L* | *Labs return (if requested) WBC 16.1 no./mm3 (90% PMNs), Hb 6.0 g/dL (baseline 6.5), platelets 585K no./mm3, Creatinine 1.4 mg/dL (bl 0.87), glucose 111 mg/dL, total bilirubin 2.8 mg/dL, direct bilirubin 1.2 mg/dL, alkaline phosphatase 147 mg/dL, AST 32 U/L, ALT 17 U/L, protein 8.5 g/dL, albumin 4.1 g/dL, Procalcitonin 0.28 ng/mL, respiratory viral panel negative, lactate 6.1 mg/dL*  *Urinalysis: negative LE, negative ketones, no WBCs* |
| *Within 5 minutes of starting the case (sooner if back not examined)* | *HR 155, BP 77/39, SpO2 88% on RA (92% on NC) patient appears pale and less responsive* | *Learner may request IVF* |
| *If learner requested intravenous fluids (****must*** *specify how much)* | *HR 140, BP 82/50 (after 1 L/hr), SpO2 88% RA (92% NC 3L)*  *HR 130, BP 90/55 (after 2L) 85% RA (90% NC 4L)* |  |
| *Learner may request to start antibiotics* | *No change* | *facilitator should prompt code leader to be specific regarding which antibiotics and rational, facilitator should prompt team to order cultures if not done* |
| *Facilitator should prompt code leader to verbalize differential for back pain and fever if not already done* | *No change* | *Ddx: epidural abscess (has some urinary retention) vs osteomyelitis/discitis vs pyelonephritis vs VOC + bacteremia from skin infection* |
| *Learner may request imaging* | *No change* | *CXR (if requested): shows mild pulmonary edema*  *Lumbar xray (if requested): shows joint space narrowing at L4, suspicious for osteomyelitis*  *MRI Lumbar spine (if requested): osteomyelitis of L2-4 with adjacent paraspinal muscle edema & inflammation, no epidural abscess seen* |
| *Within 10 minutes of starting the simulation* | *HR 145, BP 81/47, patient is more lethargic, pale appearing, less alert* | *Repeat lactate 8.5 mg/dL, Cr 1.9 mg/dL, ABG pH 7.18 CO2 30 O2 60*  *Blood cultures (if requested): return positive for GPC clusters, MecA negative (MSSA)* |
| *Learner should order vasopressors (norepinephrine)* | *(if pressors started) HR 128, BP 100/60, patient more alert and interactive*  *(if given a fluid bolus) HR 150, BP 85/52, SpO2 78% -> patient will require intubation for respiratory distress* |  |
| *Learner may call for consultation of ICU, hematology and/or orthopedics* |  |  |
| *Sign out given to MICU accepting physician* |  | *Code Leader should give a 1 liner sign out to ICU physician* |
|  |  |  |

Ideal Scenario Flow:

The learners enter the room find an acutely ill adult patient in discomfort. She has acute back pain and is feverish with early warning signs of septic shock. A full skin and thorough history are needed to make the diagnosis. The learners should identify that the patient has evidence of an infected port-a-cath on exam which has likely led to bacteremia and secondary osteomyelitis of her lumbar spine. Additionally, her symptoms of urinary retention are concerning for an epidural abscess, though not identified in this scenario, it is an important differential to mention. This patient should be judiciously volume resuscitated for her hypotension, with respect to potential right ventricular overload, given the risk of diastolic dysfunction and pulmonary hypertension in this population. Additionally, antibiotic choices should include coverage of encapsulated organisms, skin flora, and Pseudomonas given critical illness and frequent hospitalizations.

Anticipated Management Mistakes:

1. The learners may overlook the skin finding of a deeper tissue infection associated with this patient’s port-a-cath. If this detail is missed in the provided history, we recommend the facilitator prompting the team “what about her port-a-cath site?”.
2. Additionally, the learners may overlook the need to examine the patient’s back and miss the finding of lumbar tenderness. If this were to happen, we recommend the facilitator can either prompt the team to examine the back or allow the simulation to progress with the patient decompensating more rapidly.
3. The learners may continue to bolus this patient with intravenous fluids to support her blood pressures beyond what would be recommended in a patient with presumed diastolic dysfunction and right ventricular compromise. It is recommended to address this concern in the debrief session.
