# Supplementary material for: Common Complications of Sickle Cell Disease: A Simulation-Based Curriculum
Source: MedEdPORTAL. 2021 Apr 2;17:11139. doi: 10.15766/mep_2374-8265.11139 (PMC8034233; doi:10.15766/mep_2374-8265.11139)
Supplement: Supplementary file 1 — Case 1 - Acute Chest Syndrome.docxCase 2 - Stroke.docxCase 3 - Sepsis.docxSupplemental Images.docxCritical Action Checklists.docxDebrief Guide.docxPre- and Posttest.docx [file mep_2374-8265.11139-s001.zip › D. Supplemental Images.docx]

Appendix D Supplemental Images

Case 1: Acute Chest Syndrome


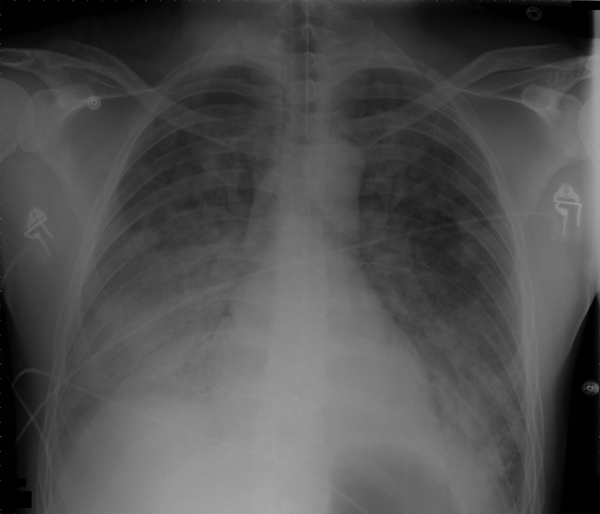


Portable Chest Radiograph demonstrating multifocal opacities

“Image by commons.wikimedia.org retrieved from:

[https://commons.wikimedia.org/wiki/File:Chest_Xray_40_yr_old_male_acute_respiratory_distress_syndrome_as_a_complication_of_murine_typhus._13-1421-F1.jpg] on [8/5/2020]. Creative Commons License associated: CC-0

Case 2: Hemorrhagic Stroke


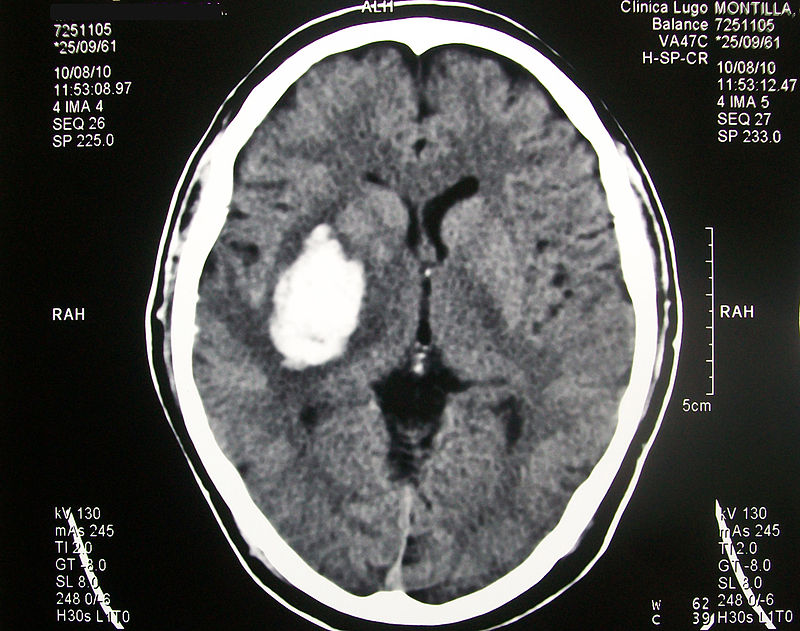


Axial non-contrast brain CT scan (a) shows intracerebral hemorrhage in the right temporal lobe (white arrow)

“Image by commons.wikimedia.org retrieved from: [http://www.pandora.com/station/play/1715099042645690453] on [7/6/2020]. Creative Commons License associated: CC BY-SA 4.0

Case 3: Sepsis in Sickle cell disease


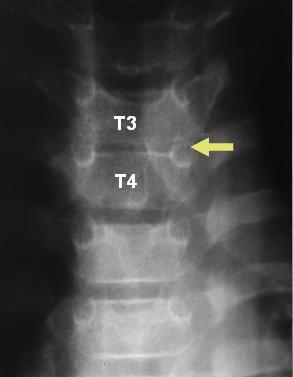


Portable radiograph with disc narrowing and reactive changes (arrow) (meant to represent lumbar spine)

“Image by commons.wikimedia.org retrieved from: [https://commons.wikimedia.org/wiki/File:Discitis_T3-T4.jpg] on [8/5/2020]. Creative Commons License associated: CC-BY-SA-4.0


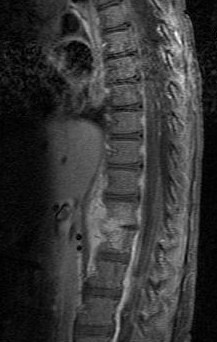


Lumbar magnetic resonance imaging demonstrating bony destruction, fat tissue stranding and edema consistent with osteomyelitis (arrow)

“Image by: commons.wikimedia.org retrieved from [https://commons.wikimedia.org/wiki/File:Discitis.jpg] on [8/5/2020]. Creative commons license associated: CC BY-SA 3.0


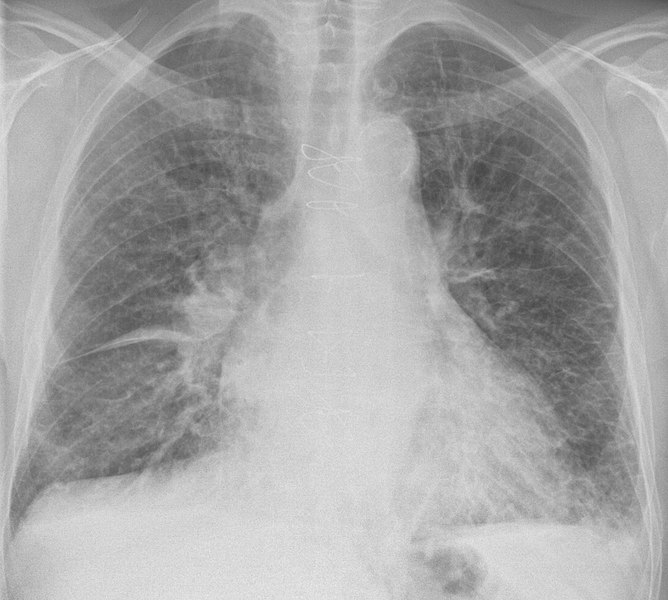


Chest radiograph demonstrating moderate pulmonary vascular congestion without evidence of focal consolidation

“Image by: commons.wikimedia.org retrieved from [https://commons.wikimedia.org/wiki/File:Chest_radiograph_of_a_lung_with_Kerley_B_lines.jpg] on [8/5/2020]. Creative commons license associated: CC-0
