# Supplementary material for: Common Complications of Sickle Cell Disease: A Simulation-Based Curriculum
Source: MedEdPORTAL. 2021 Apr 2;17:11139. doi: 10.15766/mep_2374-8265.11139 (PMC8034233; doi:10.15766/mep_2374-8265.11139)
Supplement: Supplementary file 1 — Case 1 - Acute Chest Syndrome.docxCase 2 - Stroke.docxCase 3 - Sepsis.docxSupplemental Images.docxCritical Action Checklists.docxDebrief Guide.docxPre- and Posttest.docx [file mep_2374-8265.11139-s001.zip › E. Critical Action Checklists.docx]

**Appendix E**

**Critical Action Checklists**

**Case 1 Appendix A: Acute Chest Syndrome**

- Describe an appropriate differential diagnosis for respiratory compromise
- Appropriately treat the patient with a non-rebreather
- Obtain key labs: arterial blood gas, lactate, complete blood counts, type and screen
- Obtain a portal chest radiograph and EKG
- Diagnose acute chest syndrome
- Re-assess patient, recognize hypoxia is worsening and consider non-invasive ventilation vs intubation
- Order antibiotics, describe need for coverage of atypical organisms
- Recognize need for blood transfusion (simple vs exchange transfusion; exchange will require hemodialysis catheter)
- Consider fluid balance, consider Lasix with transfusion
- Describe need for an Intensive Care Unit consult and hematology consult

**Case 2 Appendix B: Hemorrhagic Stroke**

- Describe an appropriate differential diagnosis for altered mental status
- Perform a full neurologic exam
- Obtain key labs: arterial blood gas, lactate, complete blood counts, type and screen and coagulation studies
- Recognize need for simple transfusion
- Obtain a computed tomography scan of the brain without contrast
- Diagnose hemorrhagic stroke
- Reassess patient, recognize obtundation and need for intubation
- Discuss role of reversal agents for anticoagulation
- Describe need for Intensive Care unit, hematology consult and neurosurgery consultations

**Case 3 Appendix C: Sepsis**

- Describe an appropriate differential diagnosis for back pain and fever
- Perform a head to toe exam looking for evidence of infection
- Identify bony tenderness on lumbar spine using palpation
- Obtain key labs: arterial blood gas, lactate, complete blood counts with differential, electrolytes, renal function, liver function tests, blood cultures, urinalysis and urine cultures.
- Recognize skin breakdown associated with previous port placement as a potential source of infection
- Choose broad spectrum antibiotics with coverage for encapsulated organisms
- Ensure patient has adequate access for fluid resuscitation, consider central line placement for vasopressors
- Discuss an appropriate fluid resuscitation plan recognizing likely element of diastolic dysfunction and right ventricular compromise given longstanding sickle cell disease
- Consider additional imaging to make diagnosis (ie magnetic resonance imaging of lumbar spine)
- Call for Intensive Care Unit consult given need for vasopressors
