# Supplementary material for: Common Complications of Sickle Cell Disease: A Simulation-Based Curriculum
Source: MedEdPORTAL. 2021 Apr 2;17:11139. doi: 10.15766/mep_2374-8265.11139 (PMC8034233; doi:10.15766/mep_2374-8265.11139)
Supplement: Supplementary file 1 — Case 1 - Acute Chest Syndrome.docxCase 2 - Stroke.docxCase 3 - Sepsis.docxSupplemental Images.docxCritical Action Checklists.docxDebrief Guide.docxPre- and Posttest.docx [file mep_2374-8265.11139-s001.zip › F. Debrief Guide.docx]

**Appendix F**

**Debriefing guide**

**Purpose of the debrief**

- It is a formal reflective stage in simulation learning process, in which the learner clarifies and integrates experience with previous knowledge (Decker et al., 2013)
- Debriefing is facilitated with constructive criticism; learning is reinforced and applied to real world situations through verbal dialog and reflection. (Galloway, 2009)

**Process of the debrief**

Initially address learners’ emotional responses

- Allow for discussion of how they felt (e.g., ‘How did that feel?’)
- Role of each caregiver, encourage all learners to speak
- Prioritize discussion of team communication and interactions; defer discussion of care and medicine to later in debrief
- Observe for trends and points of note

Address overall goals of scenario, focusing on points of note and areas needing improvement

- Communication goal(s)
  - The learner will practice clear communication during decompensating patient.
- Team roles goal(s)
  - The learner will learn managing team in decompensating patient scenario
- Medical goal(s); The learner will:

Discuss treatment

Discuss how to tie all goals to future practice

- How will your train of thought change the next time you encounter a similar situation?
- How would you do things differently the next time you encounter a similar situation?
- What are you taking away from your simulation experience today?

**Guided Instruction Learning Points**

Case 1: Acute Chest Syndrome

Acute chest syndrome is common in sickle cell disease. It is the second most common cause of hospitalization, affecting approximately 50% of all patients with SCDl^1.^. Vaso-occlusion within the pulmonary microvasculature is at the root of the pathophysiology of ACS. Etiologies for ACS either trigger vaso-occlusion (infection, asthma, thrombosis, hypoventilation/atelectasis) or are a result of vaso-occlusion (bone marrow and fat emboli from long bone infarction)^2^. In the National Acute Chest Syndrome Study Group, 538 adults with ACS were examined and approximately 22% required mechanical ventilation. Also in this cohort, approximately 1/5 of adults with a history of ACS developed rapidly progressing ACS associated with multisystem organ failure^3^. In moderate to severe episodes of ACS, the recommendation is to target a hemoglobin S percentage <30%. This may be best achieved using exchange transfusion, though simple transfusion can also be considered in patients who are severely anemic (Hb < 9.0 g/dl)^2^. Infections are a less common trigger of ACS in adults than children but empiric antibiotics are recommended. The most common organisms associated with ACS are viruses, atypical bacteria (*Chlamydia* and *Mycoplasma*), *Streptococcus pneumonia*and *Haemophilus influenza*. We recommend a third-generation cephalosporin along with a macrolide, or a fourth-generation fluoroquinolone as typical regimens^3^. Pain control, judicious use of fluids (avoidance of hypotension, volume overload), supplementary oxygen, bronchodilators and VTE prophylaxis are other important aspects of ACS management.

References:

1. Castro O, Brambilla DJ, Thorington B, Reindorf CA, Scott RB, Gillette P, Vera JC, Levy PS. The acute chest syndrome in sickle cell disease: incidence and risk factors. The cooperative study of sickle cell disease, Blood, 1994; 84(2):643.
2. Field JJ, DeBaun MR. Acute chest syndrome in adults with sickle cell disease, UpToDate; August 2019.
3. Vichinsky EP, Neumayr LD, Earles AN, Williams R, Lennette ET, Dean D, Nickerson B, Orringer E, McKie V, Bellevue R, Daeschner C, Manci EA. Causes and outcomes of the acute chest syndrome in adults with sickle cell disease, N Engl J Med. 2000;342(25):1855.

Case 2: Hemorrhagic Stroke

Stroke is common in sickle cell disease, it is estimated that without intervention, 11% will have a clinically apparent stroke by age 20, and 25% by age 45^1^. Ischemic stroke is more common in children and adolescents while hemorrhagic stroke is more common in young adults with SCD^2^. Peak incidence of intracranial hemorrhage occurs in those 20-29 years of age^3^. After young adulthood, ischemic strokes are more common as the more typical ischemic stroke risk factors (i.e. hypertension and diabetes mellitus) rise in incidence in combination with their progressive vasculopathy related to SCD. Cerebral hemorrhages can occur in the setting of vascular malformations termed Moyamoya or in the setting of prior ischemic infarcts^4^. An important element of management of hemorrhagic stroke is to discontinue and/or reverse any anticoagulant agents the patient may be taking. Current neurocritical care guideline recommendations include reversal of unfractionated heparin prophylaxis with protamine sulfate if the PTT is significantly prolonged^6^.

*Stroke Subtypes*

*Intracerebral hemorrhage:*

Intracerebral hemorrhage usually emanates from arterioles or small arteries. The bleeding that occurs forms a localized hematoma that spreads along white matter pathways. Accumulation of blood and neurologic symptoms occur over minutes to hours. In contrast to cerebral embolism and subarachnoid hemorrhage, the neurologic symptoms do not begin abruptly and are not maximal at onset^5^.

*Subarachnoid hemorrhage:*

Rupture of an aneurysm releases blood directly into the cerebrospinal fluid under arterial pressure. The blood spreads quickly within the cerebrospinal fluid, rapidly increasing intracranial pressure. Death or deep coma ensues if the bleeding continues. Symptoms of subarachnoid hemorrhage begin abruptly. The sudden increase in pressure causes a cessation of activity (e.g., loss of memory or focus or motor impairment). Headache is a common symptom; typically it is severe and widespread. *There are usually no focal neurologic signs* unless bleeding occurs into the brain and CSF concurrent (meningocerebral hemorrhage). Headache at the onset is typical of subarchnoid hemorrhage and this distinguishes it from other types of stroke^5^.

*Ischemic stroke:*

In SCD, this can be due to thrombosis, cerebral vasculopathy, cerebral embolism or hypo-perfusion. There may be an association with the presence of a patent foramen ovale and the occurrence of embolic strokes in patients with SCD^7^. Generally, these type of strokes present with focal neurologic findings, headache and possibly vomiting. Exchange transfusion is indicated for these patients to prevent stroke recurrence^1^.

References:

1. Lee M, Piomelli S, Granger S, Miller S, Harkness S, Brambillia D, Adams R. Stroke Prevention Trial in Sickle Cell Anemia (STOP): Extended Follow up and Final Results, Blood, 2006: 108(3): 847-851.
2. Kassim AA, GAladanci NA, Pruthi S, DeBaun MR. How I treat and manage strokes in sickle cell disease, Blood, 2015: 125(22):3401-10.
3. Ohene-frempnog K, Weiner S, Sleeper S, Miller S, Embury S, Moohr J, Wethers D, Pegelow C, Gill F. Cerebrovscular Accidents in Sickle Cell Disease: Rates and Risk Factors, Blood, 1998: 91:288-294.
4. Powars D, Adams RJ, Nichols FT, Milner P, Charache S, Sarnaik S. Delayed intracranial hemorrhage following cerebral infarction in sickle cell anemia, J Assoc Acad Minor Phys. 1990; 1(3):79.
5. Caplan, Louis R. Clinical diagnosis of stroke subtypes, UpToDate. 2019; August.
6. Frontera JA, Lewin JJ, Rabinstein AA, Aisiku IP, Alexandrov AW, Cook AM, Zoppo GJ, Kumar MA, Peerschke EIB, Stiefel MF, Teitelbaum JS, Wartenberg KE, Zerfoss CL. Guideline for reversal of antithrombotics in intracranial hemorrhage, Neurocritical Care Society and Society of Critical Care Medicine. 2015; DOI 10.1007/s12028-015-0222-x.
7. Razdan S, Strouse JJ, Naik R, et al. Patent foramen ovale in patients with sickle cell disease and stroke: case presentations and review of the literature*. Case Rep Hematol*. 2013; 2013:516705. Doi: 10.1155/2013/516705.

Case 3: Sepsis

Patients with SCD are more susceptible to infections because of functional asplenia and other immune defects. Functional asplenia increases the risk of invasive infection by encapsulated organisms, such as *Streptococcus pneumoniae*, *Haemophilus influenzae and Neisseria meningitidis^1^.* In the United States, the most common bacteria causing sepsis in SCD patients include *S. pneumoniae*, *Salmonella*, *E. coli*, and *S. aureus*. ^1^ Other important pathogens to consider in functional asplenia include *Capnocytophaga* species (dog bites), *Bordetella holmesii* (pertussis), *Babesia* species, *Plasmodium falciparum*.^2^ Both the risk and mortality associated with sepsis in asplenic patients is 2-3 fold higher compared to general popuation^2^. Empiric antibiotics should target the above pathogens and additionally be directed by local antibiogram sensitivities, A possible regimen includes Ceftriaxone (consider higher dose if high prevalence of S. pneumoniae resistance), and vancomycin (added for beta-lactamase prevalent S. pneumoniae and MRSA). If penicillin allergic, consider fluoroquinolones or meropenem. If additional immunocompromising conditions or presentation consistent with disseminated intravascular coagulation, consider intravenous immunoglobulin supplementation, though this remains controversial.^3^ It can be difficult to differentiate osteomyelitis from vaso-occlusive pain in SCD, though CRP levels can be a differentiating feature^4^. Hematogenous spread of infection is very common, areas of infarcted bone can sites of these infections and the most commonly isolated organism in adults is *S. aureus*.^5^

References:

1. Rogers, ZR. Management of fever in sickle cell disease, UpToDate, August 2019.
2. Pasternack, MS. Prevention of infection in patients with impaired splenic function, UpToDate, Aguust 2019.
3. Wang J, McQuilten ZK, Wood EM, AuBron C. Intravenous immunoglobulin in critically ill adults: when and what is the evidence? J of Critical Care; 2015(3):652
4. Fontalis A, Hughes K, Nguyen MP, Williamson M, Yeo A, Lui D, Gelfer Y. The challenge of differentiating vaso-occlusive crises from osteomyelitis in children with sickle cell disease and bone pain: A 15-year retrospective review. J Child Orthop. 2019;13(1):33.
5. Lalani T, Schmitt SK. Osteomyelitis in adults: clinical manifestations and diagnosis, UpToDate, August 2019.
