# Supplementary material for: Common Complications of Sickle Cell Disease: A Simulation-Based Curriculum
Source: MedEdPORTAL. 2021 Apr 2;17:11139. doi: 10.15766/mep_2374-8265.11139 (PMC8034233; doi:10.15766/mep_2374-8265.11139)
Supplement: Supplementary file 1 — Case 1 - Acute Chest Syndrome.docxCase 2 - Stroke.docxCase 3 - Sepsis.docxSupplemental Images.docxCritical Action Checklists.docxDebrief Guide.docxPre- and Posttest.docx [file mep_2374-8265.11139-s001.zip › G. Pre- and Posttest.docx]

Questionnaire ID: _____________________________________________

(first 2 letters of mother’s maiden name + last 4 digits of your cell phone number)

**Pre-Test / Post-Test questions**

1. I feel confident recognizing a patient who is developing acute chest syndrome.

| Not very confident | Somewhat unconfident | Neutral | Somewhat confident | Very confident |
| --- | --- | --- | --- | --- |
| 1 | 2 | 3 | 4 | 5 |

1. I feel confident identifying possible infectious etiologies of sepsis in sickle cell disease.

| Not very confident | Somewhat unconfident | Neutral | Somewhat confident | Very confident |
| --- | --- | --- | --- | --- |
| 1 | 2 | 3 | 4 | 5 |

1. I feel confident managing an acute neurologic decompensation in a patient with sickle cell disease

| Not very confident | Somewhat unconfident | Neutral | Somewhat confident | Very confident |
| --- | --- | --- | --- | --- |
| 1 | 2 | 3 | 4 | 5 |

1. What are the indications for exchange transfusion? _________________________________________

___________________________________________________________________________________

1. Name 5 common inciting factors for development of acute chest syndrome in adults?

___________________________________________________________________________________

___________________________________________________________________________________

1. What is the hemoglobin S percentage target in treatment of ACS? How can that be achieved? _________________

___________________________________________________________________________________

1. What organisms are most commonly identified in cases of ACS? _______________________

___________________________________________________________________________________

1. Strokes occur across the lifespan of patients with sickle cell disease. Hemorrhagic strokes are most common in what decade of life? (Circle correct answer)

A: 10-20 B: 20-30 C: 30-40 D: 50 and over.

1. I feel confident differentiating an acute ischemic stroke vs subarachnoid hemorrhage vs intracerebral hemorrhage based on clinical presentation.

| Not very confident | Somewhat unconfident | Neutral | Somewhat confident | Very confident |
| --- | --- | --- | --- | --- |
| 1 | 2 | 3 | 4 | 5 |

1. What is an appropriate empiric antibiotic regimen for sepsis of unknown etiology in sickle cell disease?

____________________________________________________________________________________

Answers to questions:

1. N/A
2. N/A
3. N/A
4. acute stroke, acute chest syndrome, stroke prevention
5. fat emboli from bone marrow infarction, atypical pneumonias/infection, poor ventilatory effort/atelectasis, thrombosis, CHF
6. Hb S <30%, simple vs exchange transfusion
7. atypical bacterial infections, Chlamydia pneumoniae, Mycoplasma pneumoniae, S. pneumoniae and viruses
8. B
9. N/A
10. 3^rd^ generation cephalosporin, vancomycin, discuss atypical coverage
