# Supplementary material for: Common Complications of Sickle Cell Disease: A Simulation-Based Curriculum
Source: MedEdPORTAL. 2021 Apr 2;17:11139. doi: 10.15766/mep_2374-8265.11139 (PMC8034233; doi:10.15766/mep_2374-8265.11139)
Supplement: Supplementary file 1 — Case 1 - Acute Chest Syndrome.docxCase 2 - Stroke.docxCase 3 - Sepsis.docxSupplemental Images.docxCritical Action Checklists.docxDebrief Guide.docxPre- and Posttest.docx [file mep_2374-8265.11139-s001.zip › _Educational_Summary_Report_11139.pdf]

MedEdPORTAL® The AAMC Journal of Teaching and Learning Resources

Original Publication Open Access

Common Complications of Sickle Cell Disease: A Simulation-Based Curriculum

Cassondra Cramer-Bour, MD*, Justin Peterson, MD, Barbara Walsh, MD, Elizabeth S. Klings, MD

*Corresponding author: cassondra.cramer@gmail.com

Abstract

Introduction: Sickle cell disease (SCD), the most common autosomal recessive genetic disorder worldwide, affects nearly every organ of the body and results in accelerated mortality. Nationally, internal medicine physicians lack a complete understanding of morbidity and mortality in this population leading to health care disparities. **Methods:** We created a 2-hour curriculum consisting of three SCD case vignettes representing common disease complications (acute stroke, acute chest syndrome, and septic shock) with the goal to increase medicine house staff knowledge and confidence in patient management. Residents completed a pretest to assess baseline knowledge and were divided into groups of four to five. Three simulation cases were completed by each group; learners needed to work through a differential diagnosis and describe key management steps. Each group was graded on achieving the 10 critical actions for each case. Following each case, there was a faculty-led debriefing session. Residents repeated the pretest 30 days after completion of the curriculum (posttest). **Results:** Thirty-six second year internal medicine residents participated in this curriculum. After completing this curriculum, residents improved their test score from 33% ($SD = 12\%$) to 57% ($SD = 18\%$) ($p < .0001$). Additionally, self-reported confidence in management scores increased from 2.6 ($SD = 0.8$) in the pretest to 3.5 ($SD = 0.4$) in the posttest ($p = .02$) on a 5-point Likert scale (1 = *not very confident*, 5 = *very confident*). **Discussion:** Use of a simulation curriculum increased knowledge and confidence of internal medicine residents in the management of critical illness in patients with SCD.

Keywords
Sickle Cell Disease, Anemia, Sepsis, Stroke, Acute Chest Syndrome, Critical Care Medicine, Internal Medicine, Simulation

Educational Objectives

By the end of this session, learners will be able to:

1. Identify early signs of critical illness in patients with sickle cell disease (SCD) on the inpatient service.
2. Describe clinical features of acute chest syndrome (ACS).
3. Summarize an approach to the management of ACS.
4. Evaluate the differential diagnosis of an acute neurologic decompensation in a patient with SCD.
5. Describe possible etiologies of sepsis in a patient with SCD.
6. Demonstrate leadership and teambuilding skills in the care of critically ill patients.

Citation:
Cramer-Bour C, Peterson J, Walsh B, Klings ES. Common complications of sickle cell disease: a simulation-based curriculum. *MedEdPORTAL*. 2021;17:11139.
https://doi.org/10.15766/mep_2374-8265.11139

Introduction

Sickle cell disease (SCD) is the most common autosomal recessive genetic disorder worldwide affecting 250,000-300,000 births annually. In the United States, there are 100,000 individuals living with SCD. While over 90% of children born today in the US live to adulthood, overall survival has not changed in 40 years,[1] with an average life expectancy of 54 years for persons with SCD.[2] The adult patient living with SCD often experiences fragmented health care from providers lacking in knowledge of the disease.[3] Internal medicine physicians must understand the most frequent causes of morbidity for early recognition and appropriate management to occur. However, as a rare disease in the US, many training programs do not have significant populations of patients with SCD, contributing to further health care disparities.

Adding to this problem, patient-provider communication in domains such as listening, showing respect, and spending enough time is poorer for patients with SCD compared to the general population.[4] This is likely a barrier to

Copyright © 2021 Cramer-Bour et al. This is an open-access publication distributed under the terms of the Creative Commons Attribution license.

the prompt recognition and diagnosis of SCD-related comorbidities. Numerous studies describing practitioner and patient experiences with SCD center on perceptions and treatment of vasoocclusive crises, often highlighting stigmas and misconceptions around this common presentation of SCD.[5,6]

Previous published education projects have focused on disease pathophysiology,[7] pediatric issues,[8] and cultural competency.[9] However, a gap remains in the current body of medical education literature regarding diagnosis and critical care management of complications of SCD. This curriculum was developed to improve house staff knowledge in the management of the acutely decompensating patient with SCD. We decided to develop a simulation-based curriculum to achieve this goal as simulation offers learners an immersive experience with simultaneous intercommunication skills training and critical care thinking. Simulation is an effective tool well validated for medical education.[10]

Our curriculum is based on three case vignettes highlighting common causes of morbidity and mortality in SCD: septic shock, acute chest syndrome (ACS), and strokes. First, sepsis is a major cause of hospitalization[11] in SCD and infection risk is increased due to immune defects including functional or surgical asplenia. The risk of and mortality from sepsis in asplenic patients is two- to threefold higher when compared to the general population.[12] Hyposplenia leads to deficient complement activation, rendering patients more vulnerable to encapsulated organisms (such as *Streptococcus pneumoniae* and *Haemophilis influenzae*) as well as certain viruses (such as *Parvovirus B19* and *Cytomegalovirus*).[13] Second, ACS is the second most common cause of hospitalization in SCD. However, most commonly, it presents in patients two to three days after a hospital admission for a vasoocclusive crisis. Approximately 50% of all patients with SCD will have at least one episode of ACS during their lifetime[14] and up to 22% of adults will require mechanical ventilation.[15] Despite advances in care, ACS remains a leading cause of death for SCD patients.[14] Third, acute stroke occurs across the lifespan of patients with SCD. Without risk-reducing interventions, it is estimated that 11% and 25% of SCD patients will have a clinically apparent cerebrovascular accident by ages 20 and 45, respectively.[16] Cerebral infarcts are most common in SCD patients below 20 and older than 30 years of age. During the third decade of life, hemorrhagic strokes are most common[16]; however, there is heterogeneity in stroke presentation across the lifespan and prompt accurate diagnosis is needed to guide appropriate care.

The target learner audience was second-year postgraduate internal medicine residents as they transition to the role of team leaders but could likely be adapted to target a variety of audiences. While this curriculum was developed as part of the Center for Excellence in SCD at Boston University, the largest SCD treatment center in New England treating 345 adult and 190 pediatric patients, this program can be utilized at centers with smaller populations of patients with SCD.

Methods

Development

We designed this curriculum as an educational tool to inform the management of common complications of SCD. We developed the cases assuming that all learners had a prerequisite knowledge of SCD pathophysiology. The cases were composed by a core faculty with up to 28 years of experience in caring for patients with SCD, and the material was reviewed by a panel of experts in adult and pediatric hematology, emergency medicine, and adult pulmonary/critical care. This same panel of experts collaborated together to design the critical action checklists and pre/posttest surveys. We selected faculty from the departments of medicine, pediatric emergency medicine, senior internal medicine residents, and chief residents to serve as course facilitators. This protocol was not formally reviewed by the Institutional Review Board (IRB) at Boston University School of Medicine. The rationale for this was that it was undertaken primarily for education and not for research. This was considered to be exempt research at our institution and a formal IRB application was not required.

Equipment/Environment

We used the SimMan 3G Wireless High Fidelity Human Patient Simulator (Laerdal) to run our cases, but the curriculum may be run with any simulator. Two simulators were necessary as two learner groups participated in the cases simultaneously. Each simulation was conducted in a hospital room environment which included a patient in bed with continuous cardiac monitor display. We asked participants to bring their own stethoscope. Supplies in each room included: a hospital bed, a patient mannequin, a patient gown, cardiac telemetry leads, and blood pressure cuff. Please see Appendix D for detailed descriptions of visual aids including chest radiographs pertaining to each unique case.

Personnel

The simulation session occurred weekly for 4 weeks with eight to 10 different trainees and four to five facilitators attending each session. We separated the trainees into two equal groups, each led by one technical facilitator and one faculty leader.

Copyright © 2021 Cramer-Bour et al. This is an open-access publication distributed under the terms of the Creative Commons Attribution license.

During each case, the technical facilitator was responsible for controlling the mannequin's vital signs and physical exam findings as outlined in each case summary (Appendices A-C). The faculty leader voiced responses to questions asked of the mannequin patient and provided pertinent medical history and laboratory data. As each case progressed, radiographic findings were given to the learners by the technical facilitator when requested (Appendix D). After each case concluded, we asked the two groups of learners to join together for a collective debrief session led by both faculty members (Appendix F).

Implementation
The session was 2 hours in length. An identical pre- and posttest assessment was created to test subject knowledge (Appendix G). After a brief introduction, we asked the learners to complete the pretest in under 10 minutes. The learners were divided into two groups of four to five randomly and given a printout of the clinical scenario background pertinent to that case (Appendices A-C). We asked each group to appoint one resident to serve as the team leader and for the other learners to serve as interns in each scenario. The team leader would function as the senior resident in charge of making key decisions for the management of the patient, the interns would assist with history gathering and examining the patient.

The simulations began after the team entered the simulation room. Each case simulation took 15 minutes to complete. We allowed both the team leader as well as the interns to examine the patient, ask questions regarding their symptoms and medical history, and perform physical exam maneuvers. The team leader was tasked with verbalizing a differential diagnosis and leading the management of the decompensating patient. We expected our learners to know when a specific consultation was indicated (for example, to hematology), but no additional outside help could be offered. Detailed instructions regarding the sequence of cases 1-3 can be found in Appendices A-C. Supplemental images to guide the cases can be found in Appendix D. During each simulated case, the faculty leader graded the learners' performance using the critical action checklist developed for that case (Appendix E). We asked the faculty leaders to take note of the communication style and leadership skills displayed by the team leader during the scenario. After each case, there was a 20-minute debrief led by the faculty facilitators using the debriefing guide (Appendix F). Each case took place sequentially with a different resident in the role of the team leader; see the Figure for a flow diagram of the activity organization. A voluntary posttest (Appendix G) was conducted via email 30 days after the simulation session.

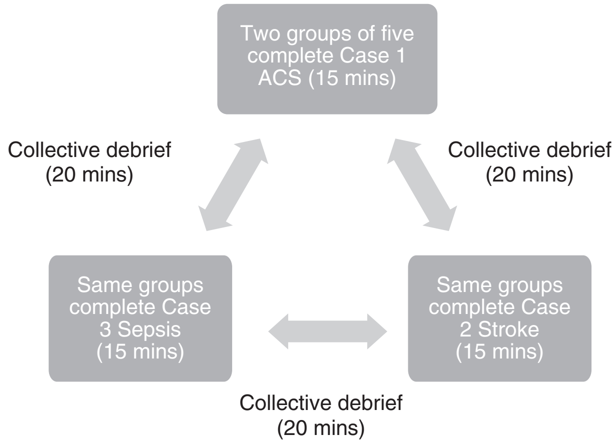

Figure. Flow diagram of the simulation activity.

Debriefing
We began each debriefing session with an open-ended question such as, "How did that feel?" This was followed by reviewing the results of the critical action checklist and discussing actions that were not completed. For example, the facilitator might say, "I see both groups knew to order broad spectrum antibiotics for treatment of severe sepsis but only one group remembered to cover encapsulated organisms." This would allow for focused discussion around the key teaching points as laid out in the critical action checklist. Though our faculty support all had experience with treating patients with SCD, we found it helpful to use the faculty teaching handout (Appendix F) to ensure consistent instruction from week to week.

Assessment
We developed the critical action checklist as a multidisciplinary team including physicians from adult pulmonary and critical care, pediatric emergency medicine, and adult and pediatric hematology (Appendix E). These checklists contained 10 items which were identified as critical items for learners to identify or perform during the management of each case and became the primary tool to frame the targeted instruction during the debrief session. Similarly, we designed the pre- and posttests (Appendix G) with the same cohort of faculty to mirror the critical action checklist items.

We evaluated the efficacy of the curriculum by administering the same pretest to the participants 30 days after the activity. The test includes six knowledge-based questions, and four Likert-scale questions (1 = *not very confident*, 5 = *very confident*) in managing patients with SCD across a variety of domains. We matched pre- and posttest scores using an anonymous

Copyright © 2021 Cramer-Bour et al. This is an open-access publication distributed under the terms of the Creative Commons Attribution license.

identifier prior to analysis. We analyzed our results by performing a Student's paired *t* test using GraphPad software and considered $p \le .05$ as significant.

In addition to the pre- and posttests, our curriculum also included a qualitative assessment of performance and leadership skills using the critical action checklist as scored by the faculty facilitator in real time. In the structured debrief time after each case scenario, our faculty leaders used this tool to provide organized feedback to the group overall. Moreover, the critical action checklist mirrored the learning objectives for each case and prompted a discussion of these details.

Results

A total of 36 PGY 2 internal medicine residents participated in this curriculum over 4 weeks. Each week, eight to 10 residents completed the activity. Twenty-two of 36 residents (61%) submitted their posttest and were considered for analysis (five submitted a posttest with a nonmatching pretest identifier). The mean pretest score was 33% (*SD* = 12%, 95% CI = 27%-38%; Table). There was a sizeable increase in the posttest score to 57% (*SD* = 18%, 95% CI = 50%-64%) which reached significance ($p = .0001$). Additionally, self-reported confidence in managing patients with SCD increased from an average of 2.5 (*SD* = 0.8, 95% CI = 2.2-2.8) on the pretest to 3.5 (*SD* = 0.4, 95% CI = 3.4-3.7) on the posttest ($p = .0176$) on a 5-point Likert scale (1 = *not very confident*, 5 = *very confident*).

In our review of the critical care action checklist scores (out of 10) for the eight groups of residents, the management of acute stroke (case 2) was the most challenging for internal medicine residents with a mean score of 6.9 (*SD* = 2.4, 95% CI = 5.2-8.5). Case 1 on ACS had a mean score of 8.3 (*SD* = 0.8, 95% CI = 7.7-8.8), whereas case 3 on sepsis had the highest level of competency with a mean score of 9.0 (*SD* = 1.6, 95% CI = 7.9-10.0). During the debriefs on the critical actions checklists, it was surprising to many residents that atypical infections are most commonly associated with the diagnosis of ACS, and it was a learning point that hemorrhagic strokes are the most commonly observed in patients 20-30 years old.

Table. Resident Results on Simulation Pre/Posttest (*N* = 22)

	Pretest		Posttest		
**Question type**	***M (SD)***	**95% CI**	***M (SD)***	**95% CI**	***p*a**
Knowledge[b]	33% (12%)	27%-38%	57% (18%)	50%-64%	.0001
Likert Scale[c]	2.5 (0.8)	2.2-2.8	3.5 (0.4)	3.4-3.7	.0176

[a]Significant at $p \le .05$.
[b]Scores reflect the percentage of correct responses on the six questions.
[c]Rated on a 5-point Likert scale (1 = *not very confident*, 5 = *very confident*).

Discussion

Patients with SCD have frequent encounters with the health care system throughout their lifespan and receive much of their care as adults by internal medicine-trained physicians. A key finding of our study was that even in centers with large SCD populations, education around SCD remains poor. It was our aim therefore to address this disparity with an immersive learning experience. Simulation was utilized to create a real-world scenario to manage a patient as a team and make treatment decisions. This process emphasized the importance of group learning and allowed for faculty-led feedback on the critical thinking applied to forming an appropriate differential diagnosis and making key management decisions.

One of the strengths of this curriculum was that it led to consolidation of knowledge as demonstrated by the nearly twofold increase in average test scores 30 days after completion of the curriculum. The greatest improvement was observed in question 4, "What are the indications for exchange transfusion?," and in question 7, "What is the hemoglobin S percentage target for ACS? How can that be achieved?" This suggests acquisition of SCD-specific knowledge. This educational activity was well received by the internal medicine house staff and there are plans to continue it annually going forward.

One of the limitations of this study included an inability to capture the faculty perception regarding team dynamics, which was a secondary aim of this exercise. During each debrief session there was an informal opportunity to discuss the importance of team communication and leadership in management of critically ill patients. This exercise may be improved in the future with a more structured rubric for team leadership comments during the simulation. Further, we were challenged by time constraints in the didactic schedule which necessitated slightly larger groups than optimal, therefore not all learners in our sessions were able to function in the team leader role. Another limitation affecting the results has to do with the staggered nature of having a small number of trainees complete the simulation each week. It was certainly possible that earlier participants could have shared their experiences of the simulation with their peers in a later group. Finally, it was observed that the participation in the posttest was low at 75% (27 of 36, though five were excluded for analysis with nonmatching identifiers) and this introduced bias that perhaps nonrespondents would have performed worse in the posttest and perhaps did not respond for this reason.

Future directions for this curriculum include incorporation with a regular rotation on the SCD service at our institution. It is

Copyright © 2021 Cramer-Bour et al. This is an open-access publication distributed under the terms of the Creative Commons Attribution license.

our hope that once the curriculum is ensconced for the PGY 2 class it can serve as not only an introduction to the team-leading responsibilities needed in a senior resident, but also a primer for anticipated complications of SCD. It would be an interesting next step to see how the posttest scores may improve if participants have immediate exposure to caring for patients with SCD. A suggestion for implementation of this curriculum at centers that do not see a high volume of patients with SCD would be to restructure the flow of cases such that the debrief periods occur after completing all three cases rather than in between, and during this 1-hour interval, bring in a guest lecturer who is an expert on SCD. Further, Appendix F contains helpful background information for facilitators with less familiarity with complications of SCD.

In conclusion, we present a simulation curriculum which was an effective tool for case-based learning in the management of patients with SCD. We hope that curricula such as this can be spread through internal medicine training programs nationally to improve clinical knowledge surrounding SCD and ultimately, improve the health care of those living with SCD.

Appendices

A. Case 1 - Acute Chest Syndrome.docx

B. Case 2 - Stroke.docx

C. Case 3 - Sepsis.docx

D. Supplemental Images.docx

E. Critical Action Checklists.docx

F. Debrief Guide.docx

G. Pre- and Posttest.docx

All appendices are peer reviewed as integral parts of the Original Publication.

Cassondra Cramer-Bour, MD: Hospitalist, Henry Ford Health Systems at Wayne State University School of Medicine; ORCID: https://orcid.org/0000-0001-5274-0666

Justin Peterson, MD: Chief Resident, Department of Internal Medicine, Boston University School of Medicine

Barbara Walsh, MD: Associate Professor, Department of Pediatrics, Boston University School of Medicine

Elizabeth S. Klings, MD: Associate Professor, Department of Medicine, Director of Center of Excellence in Sickle Cell Disease, and Director of Pulmonary Hypertension, Boston University School of Medicine; ORCID: https://orcid.org/0000-0003-4879-720X

Disclosures
None to report.

Funding/Support
None to report.

Ethical Approval
Reported as not applicable.

References

1. Chaturvedi S, DeBaun MR. Evolution of sickle cell disease from a life-threatening disease of children to a chronic disease of adults: the last 40 years. *Am J Hematol*. 2016;91(1):5-14. https://doi.org/10.1002/ajh.24235
2. Lubeck D, Agodoa I, Bhakta N, et al. Estimated life expectancy and income of patients with sickle cell disease compared with those without sickle cell disease. *JAMA*. 2019; 2(11):e1915374. https://doi.org/10.1001/jamanetworkopen.2019.15374
3. Panepinto JA, Owens PL, Mosso AL, et al. Concentration of hospital care for acute sickle cell disease-related visits. *Pediatr Blood Cancer*. 2012;59(4):685-689. https://doi.org/10.1002/pbc.24028
4. Haywood C Jr, Diener-West M, Strouse J, et al. Perceived discrimination in health care is associated with a greater burden of pain in sickle cell disease. *J Pain Symptom Manage*. 2014;48(5):934-943. https://doi.org/10.1016/j.jpainsymman.2014.02.002
5. Glassberg JA. Improving emergency department-based care of sickle cell pain. *Hematology Am Soc Hematol Educ Program*. 2017;2017(1):412-417. https://doi.org/10.1182/asheducation-2017.1.412
6. Shapiro BS, Benjamin LJ, Payne R, Heidrich G. Sickle cell-related pain: perceptions of medical practitioners. *J Pain Symptom Manage*. 1997;14(3):168-174. https://doi.org/10.1016/S0885-3924(97)00019-5
7. Hori R, Adams-Graves P, Nishimoto S, Chesney R, Brescia W. Sickle cell disease team-based learning module. *MedEdPORTAL*. 2015;11:10200. https://doi.org/10.15766/mep_2374-8265.10200
8. Lypson M, Campbell A, Stojan J, Ross P. A crash course in sickle cell disease. *MedEdPORTAL*. 2014;10:9864. https://doi.org/10.15766/mep_2374-8265.9864
9. Schwartz LA, Radcliffe J, Barakat LP. The development of a culturally sensitive pediatric pain management intervention for African American adolescents with sickle cell disease. *Child Health Care*. 2007;36(3):267-283. https://doi.org/10.1080/02739610701377954
10. Lateef F. Simulation-based learning: just like the real thing. *J Emerg Trauma Shock*. 2010;3(4):348-352. https://doi.org/10.4103/0974-2700.70743

Copyright © 2021 Cramer-Bour et al. This is an open-access publication distributed under the terms of the Creative Commons Attribution license.

11. Thi Nhat Ho A, Shmelev A, Joshi A, Ho N. Trends in hospitalizations for sickle cell disease related-complications in USA 2004-2012. *J Hematol*. 2019;8(1):11-16. https://doi.org/10.14740/jh475
12. Kristinsson SY, Gridley G, Hoover RN, Check D, Landgren O. Long-term risks after splenectomy among 8,149 cancer-free American veterans: a cohort study with up to 27 years follow-up. *Haematologica*. 2014;99(2)392-398. https://doi.org/10.3324/haematol.2013.092460
13. Castro O, Poillon WN, Finke H, Massac E. Improvement of sickle cell anemia by iron-limited erythropoiesis. *Am J Hematol*. 1994;47(2)74-81. https://doi.org/10.1002/ajh.2830470203
14. Vichinsky EP, Neumayr LD, Earles AN, et al.; National Acute Chest Syndrome Study Group. Causes and outcomes of the acute chest syndrome in sickle cell disease. *N Engl J Med*. 2000;342(25):1855-1865. https://doi.org/10.1056/NEJM200006223422502
15. Platt OS, Brambilla DJ, Rosse WF, et al. Mortality in sickle cell disease. Life expectancy and risk factors for early death. *N Engl J Med*. 1994;330(23):1639-1644. https://doi.org/10.1056/NEJM199406093302303
16. Ohene-Frempong K, Weiner SJ, Sleeper LA, et al. Cerebrovascular accidents in sickle cell disease: rates and risk factors. *Blood*. 1998;91(1):288-294.

Received: June 11, 2020
Accepted: February 1, 2021
Published: April 2, 2021

Copyright © 2021 Cramer-Bour et al. This is an open-access publication distributed under the terms of the Creative Commons Attribution license.
